# Supplementary material for: Study protocol: Dental and periodontal characteristics of older adults in Atahualpa, Ecuador within the Atahualpa Project cohort
Source: PLoS One. 2026 Mar 19;21(3):e0337166. doi: 10.1371/journal.pone.0337166 (PMC13001909; doi:10.1371/journal.pone.0337166)
Supplement: S1 File — (DOCX) [file pone.0337166.s001.docx]

**S1 File. English Questionnaires (GOHAI, HLS-EU-Q16, BHLS)**

**1. Geriatric Oral Health Assessment Index (GOHAI) – English version**

Source: Atchison KA, Dolan TA. Development of the Geriatric Oral Health Assessment Index. *J Dent Educ*. 1990.

**Instructions:**
Please indicate how often you have experienced each of the following situations during the past three months.

Response options:
1 = Always
2 = Often
3 = Sometimes
4 = Seldom
5 = Never

1. How often did you have trouble biting or chewing firm foods such as apples, meat, or corn?
2. How often did you limit the kinds or amounts of food you eat because of problems with your teeth or dentures?
3. How often were you able to swallow comfortably? *(reverse item)*
4. How often have you had discomfort or pain in your mouth?
5. How often have you been worried or concerned about problems with your teeth, gums, or dentures?
6. How often have you been self-conscious or embarrassed about your teeth, gums, or dentures?
7. How often did you feel that problems with your teeth or dentures affected your speech? *(reverse item)*
8. How often did you avoid smiling or laughing because of problems with your teeth or dentures?
9. How often did problems with your teeth or dentures interfere with your usual activities?
10. How often have you felt tense or irritable because of problems with your teeth or dentures?
11. How often did you feel that your overall life satisfaction was affected by problems with your teeth or dentures?
12. How often did you feel uncomfortable eating in front of other people because of problems with your teeth or dentures?

**2. European Health Literacy Survey Questionnaire (HLS-EU-Q16) – English version**

Source: European Health Literacy Survey (HLS-EU).

**Instructions:**
Please indicate how easy or difficult it is for you to perform the following tasks related to health.

Response options:
1 = Very difficult
2 = Difficult
3 = Easy
4 = Very easy

1. Find information about symptoms or diseases.
2. Understand what your doctor tells you.
3. Judge whether information about health in the media is reliable.
4. Make decisions about medical treatments.
5. Understand instructions from your pharmacist.
6. Judge when you need to see a doctor.
7. Find information on how to manage mental health problems.
8. Understand public health warnings (for example vaccination campaigns).
9. Follow the recommendations of your doctor or nurse.
10. Find information on how to prevent diseases.
11. Understand information on food labels.
12. Judge the health risks of behaviors such as smoking or drinking alcohol.
13. Find support to manage chronic illnesses.
14. Understand written information about your health insurance.
15. Find information about physical activity or a healthy diet.
16. Judge the reliability of health information on the Internet.

**3. Brief Health Literacy Screen (BHLS) – English version**

Source: Chew LD et al. Brief Health Literacy Screen.

**Instructions:**
Please answer the following questions about your ability to understand written health information.

Response options:
1 = Always
2 = Often
3 = Sometimes
4 = Rarely
5 = Never

1. How often do you have someone help you read hospital materials or written information from your doctor or pharmacy?
2. How often do you have problems learning about your medical condition because of difficulty understanding written information?
3. How often do you have difficulty filling out medical forms by yourself?
